# Magnetic anisotropy and GGG substrate stray field in YIG films down to millikelvin temperatures

**DOI:** 10.1038/s44306-024-00030-7

**Published:** 2024-07-02

**Authors:** Rostyslav O. Serha, Andrey A. Voronov, David Schmoll, Roman Verba, Khrystyna O. Levchenko, Sabri Koraltan, Kristýna Davídková, Barbora Budinská, Qi Wang, Oleksandr V. Dobrovolskiy, Michal Urbánek, Morris Lindner, Timmy Reimann, Carsten Dubs, Carlos Gonzalez-Ballestero, Claas Abert, Dieter Suess, Dmytro A. Bozhko, Sebastian Knauer, Andrii V. Chumak

**Affiliations:** 1 Faculty of Physics, University of Vienna, 1090 Vienna, Austria; 2 Vienna Doctoral School in Physics, University of Vienna, 1090 Vienna, Austria; 3 Institute of Magnetism, Kyiv, 03142 Ukraine; 4 Research Platform MMM Mathematics - Magnetism - Materials, University of Vienna, Vienna, Austria; 5 Huazhong University of Science and Technology, Wuhan, China; 6 CEITEC BUT, Brno University of Technology, 61200 Brno, Czech Republic; 7 INNOVENT e.V. Technologieentwicklung, 07745 Jena, Germany; 8 Institute for Theoretical Physics, Vienna University of Technology, 1040 Vienna, Austria; 9 Department of Physics and Energy Science, University of Colorado Colorado Springs, Colorado Springs, CO 80918 USA

**Keywords:** Magnetic properties and materials, Spintronics, Surfaces, interfaces and thin films

## Abstract

Quantum magnonics investigates the quantum-mechanical properties of magnons, such as quantum coherence or entanglement for solid-state quantum information technologies at the nanoscale. The most promising material for quantum magnonics is the ferrimagnetic yttrium iron garnet (YIG), which hosts magnons with the longest lifetimes. YIG films of the highest quality are grown on a paramagnetic gadolinium gallium garnet (GGG) substrate. The literature has reported that ferromagnetic resonance (FMR) frequencies of YIG/GGG decrease at temperatures below 50 K despite the increase in YIG magnetization. We investigated a 97 nm-thick YIG film grown on 500 μm-thick GGG substrate through a series of experiments conducted at temperatures as low as 30 mK, and using both analytical and numerical methods. Our findings suggest that the primary factor contributing to the FMR frequency shift is the stray magnetic field created by the partially magnetized GGG substrate. This stray field is antiparallel to the applied external field and is highly inhomogeneous, reaching up to 40 mT in the center of the sample. At temperatures below 500 mK, the GGG field exhibits a saturation that cannot be described by the standard Brillouin function for a paramagnet. Including the calculated GGG field in the analysis of the FMR frequency versus temperature dependence allowed the determination of the cubic and uniaxial anisotropies. We find that the total crystallographic anisotropy increases more than three times with the decrease in temperature down to 2 K. Our findings enable accurate predictions of the YIG/GGG magnetic systems behavior at low and ultralow millikelvin temperatures, crucial for developing quantum magnonic devices.

## Introduction

Magnonics is the field of science that deals with data carried and processed by spin waves and their quanta, magnons, in magnetically ordered media^1^. The ferrimagnet yttrium iron garnet (YIG) Y_3_Fe_5_O_12_ is the material with the lowest known magnetic damping as bulk material^2–4^ and in the form of thin films^5–11^. Thus, YIG is the medium with the longest propagation lengths and lifetimes, in which magnons exist up to one microsecond^12^. Therefore, YIG has emerged as a preeminent material in RF technologies and magnonic experiments, showing promise for quantum magnonic applications. The field of quantum magnonics is a rapidly growing and highly promising research area that operates with quantum magnonic states, e.g. single magnons, and hybrid structures^1,13–21^. These investigations have to be performed at millikelvin temperatures to ensure that there is minimal thermal noise, allowing for precise observation and manipulation of the quantum states of magnons, which are extremely fragile to any kind of distortion.

Note, YIG was already the material of choice in experiments at low kelvin and ultralow millikelvin temperature magnonics for coupling to superconducting resonators^13,18,22,23^ and propagating spin-wave spectroscopy^19,24,25^. Furthermore, the first demonstration of magnon control and detection at the single magnon level^16^ and the first measurements of the Wigner function of a single magnon^17^ were performed also using a YIG sphere as a magnetic medium. Proposals for applications in quantum computing have also emerged, in particular, the use of YIG spheres as magnonic transducers for qubits^18^. A pressing question in the quantum magnonics community is how to bring the more complex, flexible, and property-rich structures employed in classical magnonic nanodevices to the quantum regime. Two-dimensional geometries are a paramount example, for instance YIG films down to tens of nanometers thick grown on gadolinium gallium garnet (GGG) Gd_3_Ga_5_O_12_ substrates^5–8,10,11^ enable the development of nanoscale magnetic devices and circuits^1^. As the temperature decreases, the spin-wave damping in YIG/GGG increases up to tenfold due to various effects associated with impurities in YIG and parasitic influence of the paramagnetic GGG substrate^19,26–30^. Our experimental investigations of spin-wave damping agree well with the results reported in the literature (but are out of the scope of this article).

Several experimental reports have demonstrated that lowering the temperature below about 50 K in YIG/GGG shifts the ferromagnetic resonance (FMR) frequency or the frequency of propagating spin waves^25,29,31–33^. Our experimental results, discussed in this article, show the same behavior and the interpretation given by the authors in^31^, agrees with our analysis. However, at the time of the study, the measurements and calculations were not performed below 4.2 K, which is particularly interesting for quantum magnonics and is the temperature regime in which GGG shows a complex magnetic phase behavior^34,35^. Additionally, the role of the strong nonuniformity of the field induced by the partially magnetized GGG was not explored, as well as the change of crystallographic anisotropy in YIG with decreasing temperature.

Here, through experiments, theory, and numerical simulations, we studied the stray field induced by the GGG substrate at temperatures as low as 30 mK. Using vibrating-sample magnetometry (VSM), we measured the magnetization of GGG as a function of the applied field and temperature down to 1.8 K. To extrapolate values for lower temperatures, we used the Brillouin function. We utilized analytical theory and numerical simulations to determine the GGG stray field and the cubic and uniaxial crystallographic anisotropies of YIG. Our findings revealed that the GGG field is strongly nonuniform, ranging from 12 mT to 60 mT for the YIG/GGG sample with an area of (5 × 5) mm^2^, at a temperature of 1.8 K, and an applied magnetic field of 600 mT when the magnetization of GGG was 253 kA/m. It has been experimentally confirmed by FMR measurements that the magnetization of the GGG substrate does not change with temperature below 500 mK^35^ in contrast to the Brillouin model of a paramagnet. The results give access to the properties of propagating magnons in YIG/GGG at millikelvin temperatures, essential for the future of nanoscale quantum-magnonic circuits.

## Results and discussion

**Fig. 1:**
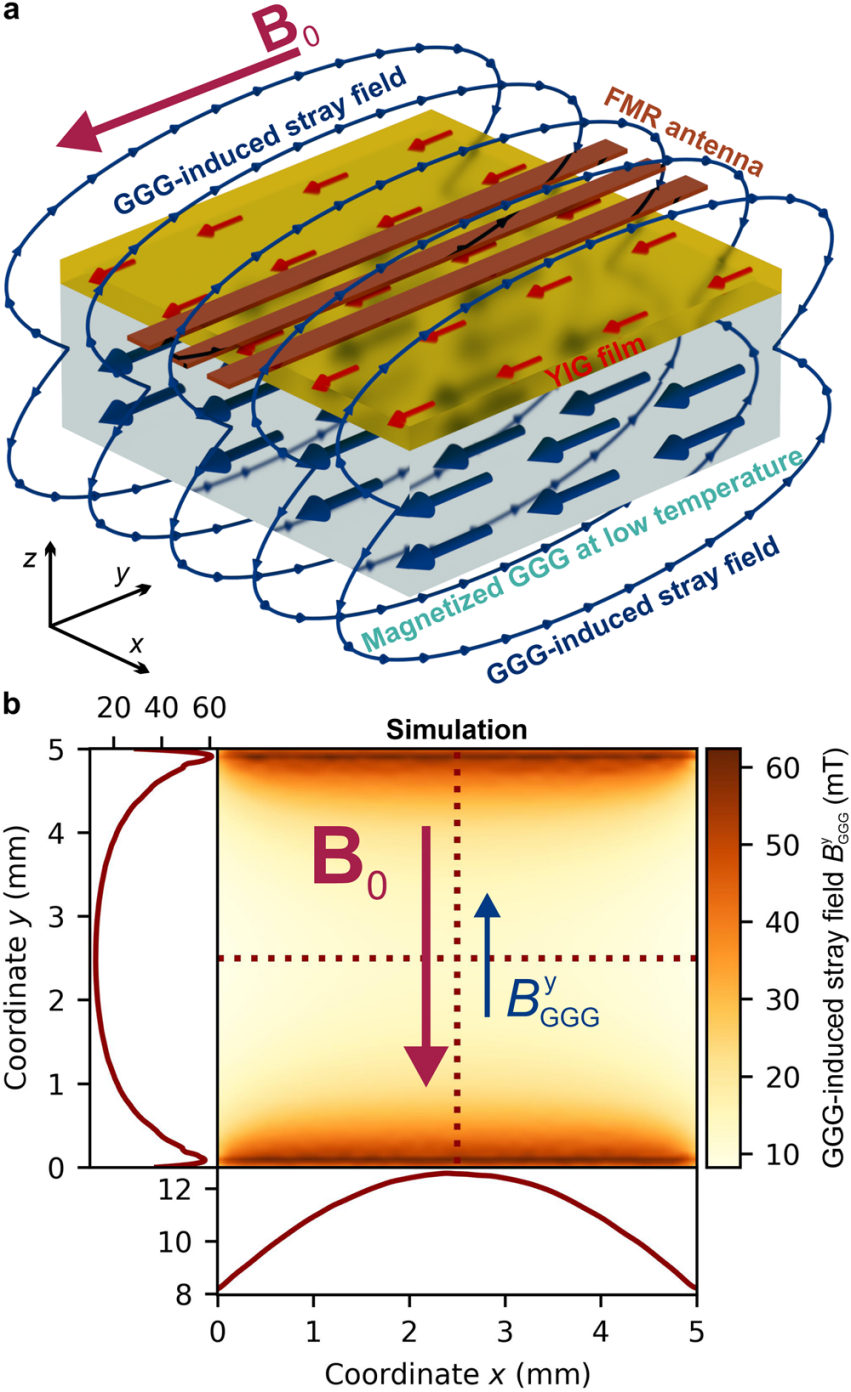
GGG-induced magnetic stray field. **a** Depiction of the experimental system of a YIG film grown on GGG. The sample is in-plane magnetized by an external magnetic field, and at temperatures approaching 0 K the paramagnetic GGG spin system saturates. The substrate creates an inhomogeneous stray field, that becomes an additional component to the internal magnetic field of the YIG but is oriented antiparallel to the external field. To measure FMR the YIG film sample is placed on a CPW antenna, through which the magnetic system is excited. **b** Simulation of the highly inhomogeneous ${B}_{{{{\rm{GGG}}}}}^{{{{\rm{y}}}}}$ stray field y-component at the interface between YIG and GGG layers. The inhomogeneity can also be seen for the *x* and *y*-axis for the center of the sample by the two cross-section plots marked in the 2D map with the red dotted lines. The simulation is performed using a non-linear Maxwell solver^47^ for the temperature 1.8 K and the strength of the applied external magnetic field of 600 mT at which the magnetization of GGG was 253 kA/m.

**Fig. 2:**
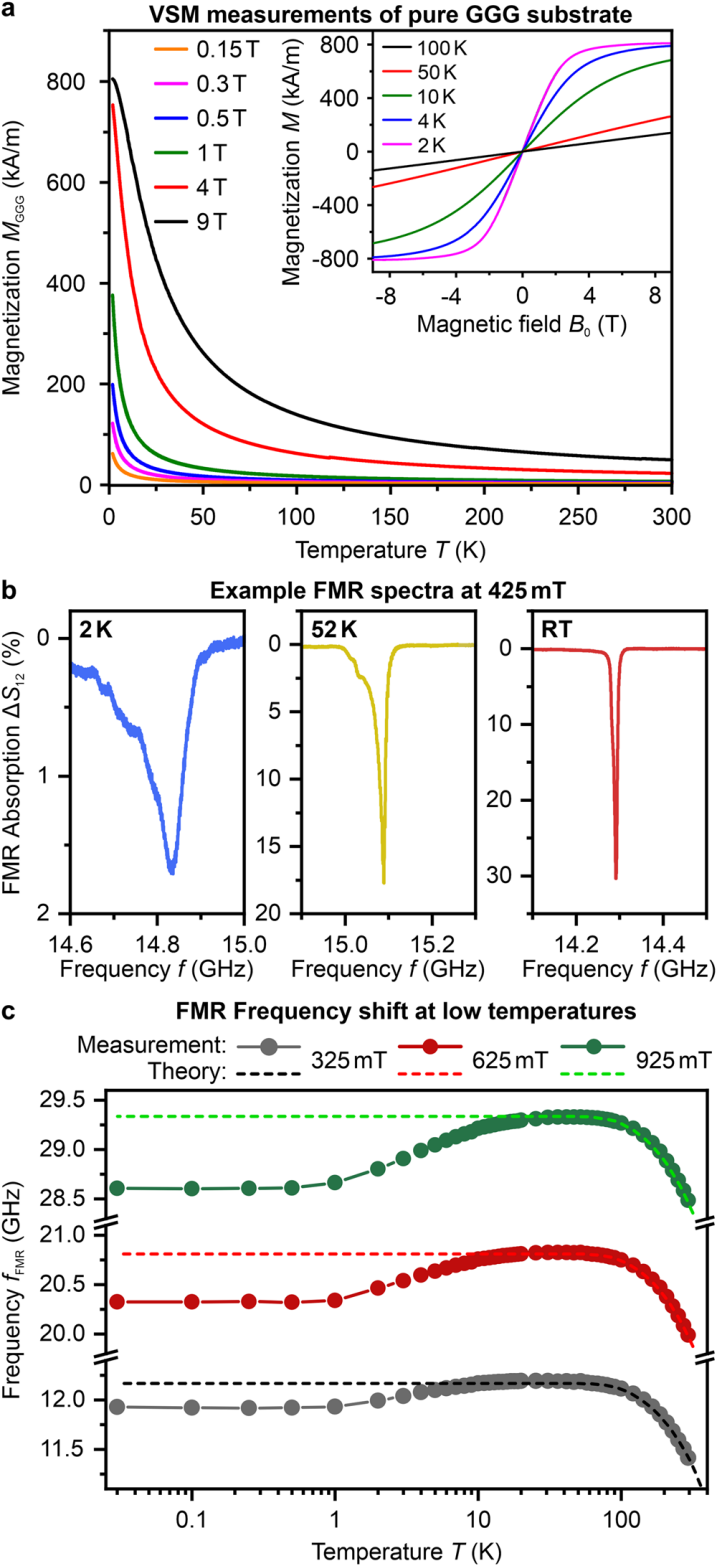
VSM and FMR measurements. **a** VSM measurements of the magnetization of GGG *M*_GGG_ as a function of the temperature *T* and the external magnetic field *B*_0_. The used sample is a (3 × 3 × 0.5) mm^3^ monocrystaline GGG slab, magnetized along one of the long axes. *M*_GGG_ saturates at a value of 805 kA/m. The graphs represent the GGG magnetization over the range available in the experiment. **b** Example spectra of FMR at the temperatures of 2 K, 52 K, and room temperature (RT) for an external magnetic field of 425 mT. **c** FMR frequency as function over the temperature, plotted in a *x*-axis logarithmic scale, for three different magnetic fields. Measurements are depicted as points and were performed with the sample magnetized in the $\langle 1\overline{1}0\rangle$ direction. The dotted lines are portraying the analytical calculation for the frequency of the FMR by the Kittel formula (Eq. (4)), which is neglecting the induced GGG stray field (*B*_GGG_ = 0). The theoretical values for ${M}_{{{{\rm{YIG}}}}}^{{{{\rm{s}}}}}$ are taken from^44^. The parameters for the gyromagnetic ratio *γ* and effective anisotropy field ${B}_{{{{\rm{ani}}}}}^{{{{\rm{eff}}}}}$ are obtained by fitting room temperature measurements.

To simulate the strength and profile of the stray field induced by the paramagnetic substrate over the YIG layer, we first measured the magnetization of a bare GGG substrate, as shown in Fig. 2a. In the partially magnetized state, GGG has a small value of magnetic susceptibility *χ* ≈ 0.3 and creates a highly inhomogeneous y-field component ${B}_{{{{\rm{GGG}}}}}^{{{{\rm{y}}}}}$ on its surface, as seen in Fig. 1b. As an example, at 1.8 K, this induced field ${B}_{{{{\rm{GGG}}}}}^{{{{\rm{y}}}}}$ opposes the external magnetic field of 600 mT. It varies in strength from 12 mT at the center to 60 mT at the edges, antiparallel to the direction of the external magnetic field. This field is crucial in investigating YIG/GGG systems at low temperatures.

Figure 2b displays two spectra that exemplify the impact of cryogenic temperatures on the FMR signal of the YIG film in an external magnetic field of 425 mT. The FMR peak is almost entirely Lorentzian-shaped at room temperature. However, when the temperature decreases below 100 K, the peak becomes asymmetric on one side, as shown in Fig. 2b for 52 K, and then broadens significantly with a lower resonance frequency, as demonstrated for 2 K. Additionally, the amplitude of the FMR peak decreases as the temperature reduces.

Figure 2c clearly shows the impact of the stray field induced by the GGG substrate. The graph displays the FMR frequencies *f*_FMR_ obtained for the YIG film at various temperatures, ranging from 300 K to 30 mK. As the temperature decreases, the FMR frequency increases due to the rise in the saturation magnetization of YIG. The theoretical curves, represented by dashed lines, were calculated using Eq. (4), with the gyromagnetic ratio *γ* and effective anisotropy field ${B}_{{{{\rm{ani}}}}}^{{{{\rm{eff}}}}}$ taken from the Kittel fit at room temperature, but neglecting the contribution of the GGG-induced stray field (*B*_GGG_ = 0). The theoretically expected FMR frequency saturates at temperatures below 50 K.

At temperatures below 50 K, the experimental FMR frequencies deviate from the theoretical values due to the GGG-induced stray field. The data indicate that for an external field *B*_0_ of 925 mT, the FMR frequency begins to decrease at approximately 50 K, while for *B*_0_ values of 325 mT, this occurs at around 25 K. At temperatures below 2 K and high external fields of 925 mT, there is a notable deviation of over 0.5 GHz between the experimental and theoretical results. This difference is attributed to the GGG stray field, which opposes the external magnetic field and lowers the FMR frequency *f*_FMR_ of YIG. The FMR frequency shift, dependent on *M*_GGG_, becomes more pronounced as the temperature decreases and the external magnetic field increases, as shown in Fig. 2c. The results of comparing three different magnetic fields at 30 mK shows that the impact is more pronounced at higher excitation frequencies, which is associated with stronger magnetic fields.

**Fig. 3:**
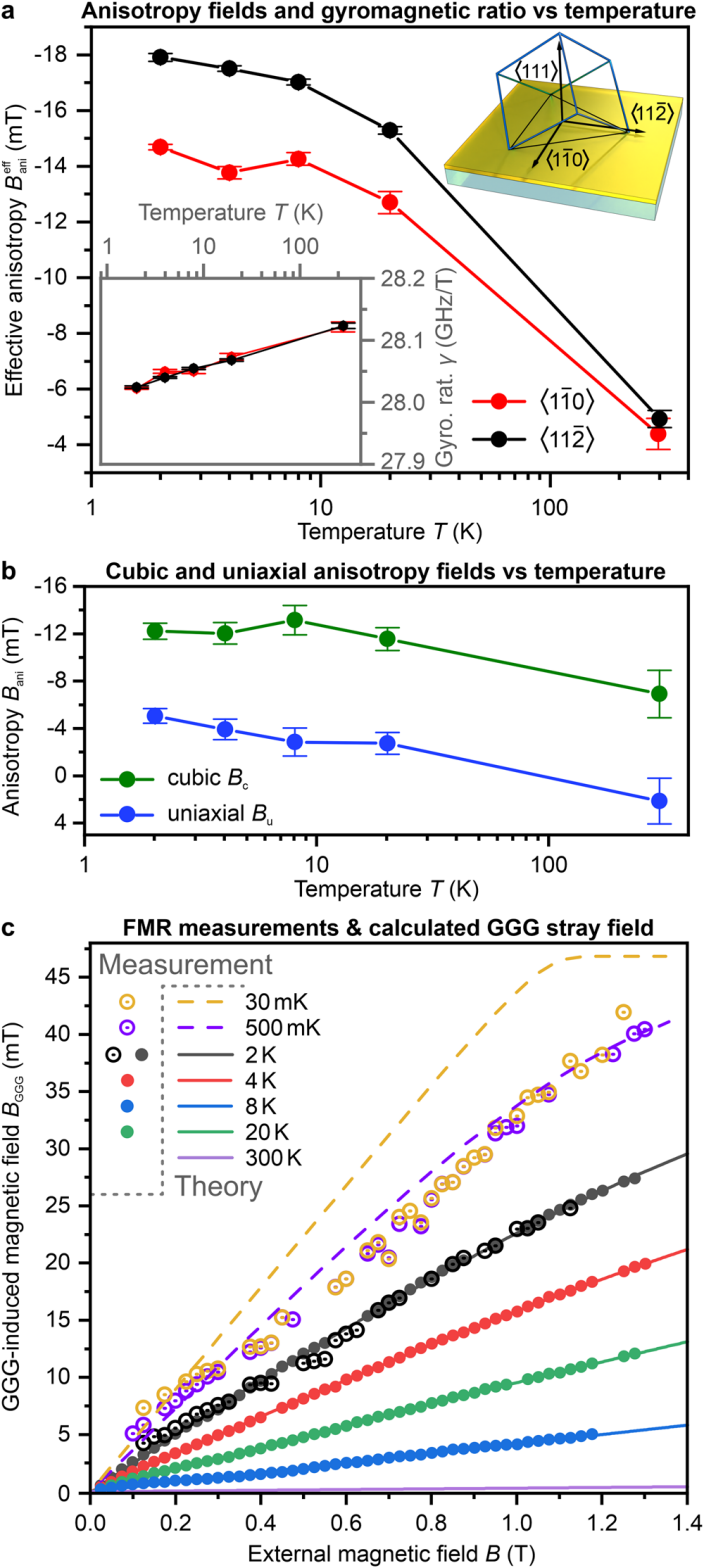
Crystallographic anisotropies and magnitude of GGG-induced stray field. **a** Effective anisotropy field ${B}_{{{{\rm{ani}}}}}^{{{{\rm{eff}}}}}$ of the YIG film as a function of the temperature in a *x*-axis logarithmic scale for two different crystallographic magnetization directions — $\langle 1\overline{1}0\rangle$ and $\langle 11\overline{2}\rangle$, schematically shown by the inset in the right corner. The inset in the left corner depicts the gyromagnetic ratio *γ* as a function of the temperature for the same magnetization geometries respectively. The points are obtained as fitting parameters from Eq. (4). **b** Cubic and uniaxial anisotropiy fields *B*_c_, *B*_u_ vs temperature *T*. Values for *B*_c_ and *B*_u_ were obtained from according Eq. (1) and (2) for the Kittel fits^40^. **c** GGG-induced stray field in YIG film center as a function of the externally applied magnetic field *B*_0_. Solid lines are the values calculated from the measured *M*_GGG_ by Eq. (5) of the VSM and solid lines are values calculated from the FMR peaks measured in the PPMS by converting Eq. (4) for *B*_GGG_. The hollow points are *B*_GGG_ values calculated from FMR peak measurements performed in the dilution refrigerator. The dotted lines are calculated via Eq. (5) by taking the extrapolated values of *M*_GGG_ by the Fit from Eq. (6).

Below 500 mK, the frequency *f*_FMR_ displays unusual behavior as its decline stabilizes, showing negligible change down to 30 mK, despite the varied magnetization of GGG in this temperature range as predicted by the Brillouin function (Eq. (6)). This phenomenon is due to the complex nature of GGG, which possesses a geometrically highly frustrated spin system^36,37^, leading to a complex phase diagram for temperatures below 1 K^34,35^. Understanding the sub-kelvin temperature behavior of GGG requires considering competing interactions among loops of spins, trimers, and decagons, along with the interplay between antiferromagnetic, incommensurate, and ferromagnetic orders^35^. Consequently, the Brillouin function fails to describe the magnetization of GGG below 500 mK and can be seen even clearer in later described Fig. 3c. This finding aligns with previous experimental studies^35^ using different techniques, such as single-crystal magnetometry and polarized neutron diffraction. Both a single crystal and a powder GGG sample were investigated in this study and showed the same behavior.

Incorporating the analytically calculated GGG stray field *B*_GGG_ into the Kittel formula Eq. (4) is necessary for determining the FMR frequency. To accurately identify the FMR frequencies *f*_FMR_, e.g. shown in Fig. 2b, it is essential to account for the GGG-induced stray field *B*_GGG_ at the sample center. At this location, the gradient is zero, and the region of the same field magnitude is the largest, most significantly affecting the area excited by the microwave stripline and determining the position of the FMR peak. The red vertical dashed line in Fig. 1b approximately depicts the position of the microstrip FMR antenna. Once incorporated into the Kittel equation, the gyromagnetic ratio *γ* and the effective anisotropy field ${B}_{{{{\rm{ani}}}}}^{{{{\rm{eff}}}}}$ become fitting parameters. Obtaining ${B}_{{{{\rm{ani}}}}}^{{{{\rm{eff}}}}}$ allows us to gain insight into and make predictions about the internal field of thin YIG films at low temperatures. Figure 3a displays the fitting outcomes for the magnetization orientations of $\langle 1\overline{1}0\rangle$ (red) and $\langle 11\overline{2}\rangle$ (black), with the effective anisotropy field ${B}_{{{{\rm{ani}}}}}^{{{{\rm{eff}}}}}$ plotted against temperature, with the *x*-axis in a logarithmic scale. The error bars are taken from the root-mean-square deviation of the fit. At room temperature, the anisotropy field is relatively small, measuring approximately 5 mT, with a variation of about 0.6 mT between the two orientations due to the cubic anisotropy of the YIG single crystal.

However, as the temperature decreases, both the strength of ${B}_{{{{\rm{ani}}}}}^{{{{\rm{eff}}}}}$ and the difference between $\langle 1\overline{1}0\rangle$ and $\langle 11\overline{2}\rangle$ magnetization directions increase significantly. At a temperature of 2 K, the effective anisotropy field ${B}_{{{{\rm{ani}}}}}^{{{{\rm{eff}}}}}$ is more than 3.2 and 3.5 times larger for the $\langle 1\overline{1}0\rangle$ and $\langle 11\overline{2}\rangle$ directions, respectively, than at room temperature. The inset in Fig. 3a displays the second fitting parameter, the gyromagnetic ratio *γ*, as a function of temperature on a *x*-axis logarithmic scale. It is known from previous research on YIG that *γ* is considered to be weakly temperature dependent^30,38,39^. The behavior of *γ* shows a very weak decrease at lower temperatures, changing from 28.13 GHz/T to 28.02 GHz/T. *γ* is nearly identical for both magnetization directions, and the values fall within each other’s error bars.

To get a better understanding of the changes in the effective anisotropy field ${B}_{{{{\rm{ani}}}}}^{{{{\rm{eff}}}}}$, the field was divided into its two main components: the cubic anisotropy field *B*_c_ and the uniaxial anisotropy field *B*_u_. This separation is achieved by considering the two magnetization directions and applying two separate equations for the FMR frequency, as described in^40^.

1 $${f}_{{{{\rm{FMR}}}}}^{\langle 1\overline{1}0\rangle }={\gamma }^{\langle 1\overline{1}0\rangle }\cdot \sqrt{({B}_{0}-{B}_{{{{\rm{GGG}}}}})\cdot ({B}_{0}-{B}_{{{{\rm{GGG}}}}}-{B}_{{{{\rm{u}}}}}-{B}_{{{{\rm{c}}}}}+{\mu }_{0}\,{M}_{{{{\rm{s}}}}})-2{B}_{{{{\rm{c}}}}}^{2}},$$

2 $${f}_{{{{\rm{FMR}}}}}^{\langle 11\overline{2}\rangle }={\gamma }^{\langle 11\overline{2}\rangle }\cdot \sqrt{({B}_{0}-{B}_{{{{\rm{GGG}}}}})\cdot ({B}_{0}-{B}_{{{{\rm{GGG}}}}}-{B}_{{{{\rm{u}}}}}-{B}_{{{{\rm{c}}}}}+{\mu }_{0}\,{M}_{{{{\rm{s}}}}})}.$$

Using Eq. (1) and Eq. (2) to fit the FMR frequency *f*_FMR_ data, and utilizing the gamma values from Fig. 3a, we can obtain values for the two distinct anisotropy fields, *B*_c_ (green) and *B*_u_ (blue) and their errors by root-mean-square deviation. The resulting plot in Fig. 3b shows the anisotropy field *B*_ani_ as a function of temperature *T* on a *x*-axis logarithmic scale. Our experimental findings at room temperature indicate (−6.9 ± 2) mT for *B*_c_ and (2.1 ± 2) mT for *B*_u_, which agree well with previously reported values for thin YIG films^6^. Notably, the cubic anisotropy increases to (−12.2 ± 0.8) mT at temperatures as low as 2 K, almost doubling the room temperature value. The uniaxial anisotropy exhibits a unique characteristic of changing its positive-to-negative sign at cryogenic temperatures and reaching a peak value of (−5.1 ± 0.7) mT at 2 K.

With this understanding of the low-temperature anisotropy and the stray field caused by GGG, we can make accurate predictions about the FMR behavior in YIG films even at temperatures as low as 2 K. Note, that the anisotropy increase reaches a saturation point below 10 K, as demonstrated in Fig. 3a. As a result, we can assume that the anisotropy remains constant down to the millikelvin temperatures and can be treated as equivalent to the 2 K values.

However, in the absence of magnetization *M*_GGG_ measurements below 2 K, we cannot utilize experimental data to compute the stray field *B*_GGG_ using Eq. (5). Therefore, we must rely on the Brillouin fit presented in Sec. III-A (Eq. (6)) and extrapolate *M*_GGG_ as an estimation. Additionally, a second method was used to determine the values for *B*_GGG_ at millikelvin temperatures in the center of the YIG sample by measuring the FMR position at these temperatures and rearranging Eq. (4) to solve for *B*_GGG_.

The results are shown in Fig. 3c. It depicts the stray field induced by GGG at the center of the sample as a function of the externally applied magnetic field *B*_0_. The solid lines represent the calculated values of *B*_GGG_ for temperatures of 2 K and above, while the solid points represent the obtained *B*_GGG_ from the FMR measurements. Both data sets match perfectly. This alignment, in Eq. (4), highlights the accuracy of the fitted effective anisotropy ${B}_{{{{\rm{ani}}}}}^{{{{\rm{eff}}}}}$, affirming the precision and appropriateness of the fit. The data points shown by the hollow-center-dot illustrate the *B*_GGG_ values acquired via FMR measurements in the dilution refrigerator, spanning a temperature range of 30 mK to 2 K. It is worth noting that the values of *B*_GGG_ at 2 K obtained from both the dilution refrigerator and the PPMS measurements are consistent.

The measurements within the dilution refrigerator exhibited a lower signal-to-noise ratio, which led to a higher scattering of the data points in Fig. 3c. At temperatures of 2 K, 500 mK, and 30 mK, it was necessary to maintain the applied power at a low level (below -25 dBm) to ensure that the system remained in thermal equilibrium, which resulted in a more noisy signal. This more noisy signal, together with the asymmetry of the FMR signal (see Fig. 2b at 2 K), increased the uncertainty of the FMR frequency fit (see Methods below).

The extrapolated data obtained through the Brillouin fit is shown as dotted lines for both 500 mK and 30 mK, in comparison to the experimental data (Fig. 3c). At 500 mK, the extrapolation matches at fields above 1 T and below 300 mT with the experimental data but diverts in between (purple circle points and dashed line). At 30 mK, it does not match (yellow circle points and dashed line). The extrapolated field strength of GGG (dashed yellow) experiences a sharp increase and then saturates above 1.1 T. While the extrapolated curves deviate, the measured data for 30 mK and 500 mK overlap. These results show that the method of fitting the GGG magnetization *M*_GGG_ and the stray magnetic field *B*_GGG_ effectively describes the inter- and extrapolations only at temperatures above 500 mK. This limitation confirms the conclusion above. *M*_GGG_ is solely dependent on the externally applied magnetic field below 500 mK, which is supported by the behavior of the FMR frequency in Fig. 2c and previous research on the complex behavior of GGG phase states^35^. At these temperatures, GGG was observed to transition through various magnetic phases, such as spin glass and antiferromagnetic, depending on the external magnetic field^34^.

To conclude, our findings demonstrate that YIG films grown on GGG substrates are impacted by stray fields originating from the partially magnetized paramagnetic GGG at low temperatures and under externally applied magnetic fields. The strength and configuration of these fields depend on the shape of the GGG substrate (the ratio between the width, length and thickness of the sample) and are highly inhomogeneous across the YIG layer. In the in-plane magnetization geometry, the stray field can reach up to 40 mT in the center and increases five-fold at the edges of the sample. Taking into account this geometrical behavior, one can change the shape of the substrate and thus keep the magnetic field homogeneous over the surface area, for example, in quantum-magnonic circuits based on YIG/GGG structures.

We used an analytical approach validated by micromagnetic simulations to calculate the stray field *B*_GGG_ induced by GGG. This approach allowed us to integrate *B*_GGG_ into the Kittel-fit formula and accurately determine the effective anisotropy field ${B}_{{{{\rm{ani}}}}}^{{{{\rm{eff}}}}}$ in the crystallographic directions $\langle 1\overline{1}0\rangle$ and $\langle 11\overline{2}\rangle$ of the YIG film for temperatures as low as 2 K. Moreover, we were able to extract the crystallographic cubic and uniaxial anisotropy fields, *B*_c_ and *B*_u_, respectively. These fields increase in magnitude from −6.9 mT and 2.1 mT at room temperature to −12.2 mT and −5.1 mT at 2 K.

The anomalous behavior of the FMR frequency of YIG, which is constant for temperatures below 500 mK, can be explained by the absence of the variation of the GGG magnetization *M*_GGG_ with decreasing temperature, and therefore by the GGG-induced magnetic field *B*_GGG_. This behavior can be described by the property of GGG as a geometrically highly frustrated magnet, resulting in the complex phase transition diagram of GGG at these temperatures and fields^34–37^. Our results allow accurate predictions of the YIG/GGG magnetic behavior at low and ultralow temperatures, a key element for successfully implementing future YIG/GGG quantum-magnonic networks. Additionally, the phenomena generally apply to any magnetic thin film grown on any paramagnetic substrate at low temperatures in the presence of a magnetic field.

## Methods

### Experimental methods

In our research, we studied a (5 × 5) mm^2^ and 97 nm-thick YIG film grown on a 500 μm-thick GGG substrate, using liquid phase epitaxy^5,6^. We conducted stripline ferromagnetic-resonance (FMR) spectroscopy using a vector network analyzer (VNA), within a Physical Property Measurement System (PPMS), at temperatures ranging from 2 K to 300 K and up to 40 GHz. The sample was mounted on a frequency-broadband stripline and placed in a homogeneous magnetic field of up to 1.3 T created by superconducting coils. The maximum applied microwave power in the PPMS was -5 dBm. Ultralow temperature measurements were performed in a dilution refrigerator, reaching base temperatures below 10 mK. At 20 mK, the cooling power is 14 μW, sufficient to keep the system in thermal equilibrium during the FMR spectroscopy measurements at -25 dBm applied power.

The following measurements were taken with the external magnetic field in the in-plane orientation and applied along the FMR stripline antenna. To determine the FMR spectrum for a specific field, *S*_12_ and *S*_21_ parameters were measured using a VNA not only at the target field but also at reference fields adjusted to approximately 15 mT to 40 mT, both above and below the desired value^41^. By subtracting the averaged signals of the reference fields from the measured FMR signal, we obtained the FMR absorption spectrum in YIG that was not affected by GGG (see Fig. 2b as an example). This dual reference measurement approach enabled to obtain the best results when working with kelvin and sub-kelvin temperatures, since the GGG parasitic signal is greatly affected by the change in the applied field. To obtain the resonance frequency and full linewidth at half maximum, the background is first analyzed using a 1D cubic spline model^42^. The resonance shape is then fitted using the double Lorentzian model, which individually describes the left and right sides of the asymmetric absorption peaks.

To accurately determine the magnetization of GGG for our analytical calculations and numerical simulations, we utilized vibrating-sample magnetometry (VSM) on a pure GGG slab (3 × 3 × 0.5) mm^3^ in the temperature range from 1.8 K to 300 K. The raw measurement VSM data is dependent on the sample shape due to self-demagnetization. Shape-independent values of the magnetization of GGG were extracted by calculating the internal magnetic fields *B*_int_ by

3 $${B}_{{{{\rm{int}}}}}={B}_{{{{\rm{0}}}}}-{\mu }_{0}{M}_{{{{\rm{GGG}}}}}\cdot N_{0},$$

where *B*_0_ is the externally applied magnetic field, *M*_GGG_ the measured magnetization and *N*_0_ the demagnetization factor corresponding to the magnetization direction. The Gd^+3^ ions in GGG have a relatively large spin (S = 7/2), resulting in a saturation magnetization that is notably higher than that of YIG. Specifically, the saturation magnetization of GGG, denoted by ${M}_{{{{\rm{GGG}}}}}^{{{{\rm{s}}}}}$, is equal to 805 kA/m, as shown in Fig. 2a.

Based on established practices, experimental FMR data can be used to extract the effective magnetization, the anisotropy fields, and the Gilbert damping parameter of a magnetic material (see ref. ^43^ and the supplementary materials therein). In this study, the temperature-dependent saturation magnetization of YIG, ${M}_{{{{\rm{YIG}}}}}^{{{{\rm{s}}}}}$, is taken from the analytical calculation performed in^44^. Thus, we can use this information to more accurately determine the anisotropy fields of YIG using them as a fitting parameter.

As reported in the literature^19,31^ and supported by our FMR analysis, the paramagnetic GGG becomes sufficiently magnetized at temperatures below about 100 K, together with an external magnetic field applied. This magnetization induces a magnetic stray field *B*_GGG_ in the YIG layer, which causes a shift of the YIG FMR frequencies^31^. For the in-plane applied magnetic field, the FMR shift is toward lower frequencies because *B*_GGG_ and the applied bias field *B*_0_ are antiparallel. Conversely, in an out-of-plane geometry, the stray field *B*_GGG_ aligns parallel to the field *B*_0_, resulting in a shift of the resonance frequency to higher values^31^. The positive shift was also confirmed by our experimental results, but in this paper, we focus only on the in-plane configuration.

The magnitude of this inhomogeneous stray field *B*_GGG_ is influenced by both the temperature and the strength of the external magnetic field. At lower temperatures and higher external fields, the GGG-induced stray field becomes more pronounced, which is crucial for determining the FMR frequency *f*_FMR_. The modified Kittel formula tailored for in-plane magnetization geometry describes the influence of this field on the FMR frequency:

4 $${f}_{{{{\rm{FMR}}}}}=\gamma \cdot \sqrt{({B}_{0}-{B}_{{{{\rm{GGG}}}}})\cdot ({B}_{0}-{B}_{{{{\rm{GGG}}}}}+{B}_{{{{\rm{ani}}}}}^{{{{\rm{eff}}}}}+{\mu }_{0}\,{M}_{{{{\rm{YIG}}}}}^{{{{\rm{s}}}}})},$$

where *γ* is the reduced gyromagnetic ratio, *B*_0_ is the applied external field, ${B}_{{{{\rm{ani}}}}}^{{{{\rm{eff}}}}}$ the effective crystallographic anisotropy field^43^, which is a fit parameter as described in detail below, *B*_GGG_ is the GGG-induced stray field defined analytically in the next section and ${M}_{{{{\rm{YIG}}}}}^{{{{\rm{s}}}}}$ the theoretical value for the saturation magnetization of YIG at any temperature taken from^44^.

### Analytical calculation of GGG field

At low temperatures below 10 K and in the presence of magnetic fields measuring several hundred millitesla, GGG attains a significant magnetization exceeding hundreds of kA/m (see Fig. 2a). In this state, the GGG substrate becomes a magnet and emits a stray magnetic field that expands beyond its volume. To accurately determine the strength and characteristics of the stray field generated by the GGG, two crucial parameters are required: *M*_GGG_, which denotes the magnetization of GGG, and ${\tilde{N}}_{xx}$, which represents the averaged mutual in-plane demagnetization factor of GGG and YIG layers. Then, one can accurately determine the strength and characteristics of the stray field generated by the GGG in the direction of the external field *B*_GGG_ as

5 $${B}_{{{{\rm{GGG}}}}}={\mu }_{0}\,{M}_{{{{\rm{GGG}}}}}(T,{B}_{0})\,{\tilde{N}}_{xx}.$$

*B*_GGG_ is crucial for understanding its overall magnetic influence, particularly at low temperatures, on the internal field of the YIG film. Net GGG magnetization *M*_GGG_ is given by the implicit equation^45^:

6 $${M}_{{{{\rm{GGG}}}}}={M}_{{{{\rm{GGG}}}}}^{{{{\rm{s}}}}}\cdot {{{{\mathscr{B}}}}}_{\frac{7}{2}}\left(\frac{7}{2}\cdot \frac{g\,{\mu }_{{{{\rm{B}}}}}\cdot ({B}_{0}-{\mu }_{0}\,{M}_{{{{\rm{GGG}}}}}\,{N}_{xx}+\lambda \,{\mu }_{0}\,{M}_{{{{\rm{GGG}}}}})}{{k}_{{{{\rm{B}}}}}\,T}\right),$$

where ${M}_{{{{\rm{GGG}}}}}^{{{{\rm{s}}}}}$ = 805 kA/m denotes the saturation magnetization of GGG, *g* = 2 the Landé factor, *μ*_B_ the Bohr magneton, ${{{{\mathscr{B}}}}}_{\frac{7}{2}}$ the Brillouin function for the angular momentum *J* =$\,\frac{7}{2}$, *λ* the coefficient of the molecular field and *N*_*xx*_ is the standard demagnetization factor (i.e. self-demagnetization) of the YIG/GGG sample.

In our case of a thin GGG cuboid of the sizes 2*a* × 2*a* × 2*c*, where *c* ≪ *a*, the self-demagnetization factor can be approximated as^46^

7 $${N}_{xx}\,\approx\, \frac{c}{\pi a}\left(0.726-\log \frac{c}{a}\right).$$

For the mutual demagnetization factor, we account that the YIG film is much thinner than the GGG substrate, and that we deal with nonuniform FMR in YIG, because the lateral sizes of YIG are much larger than the spin-wave free path, so that standing eigenmodes are not formed. In this case, the FMR peak position is mostly determined by the central YIG area, for which the mutual demagnetization factor is

8 $${\tilde{N}}_{xx}=\frac{1}{\pi }\cdot \arctan \left[\frac{\sqrt{2}c}{\sqrt{{a}^{2}+2{c}^{2}}}\right].$$

Full expressions for arbitrary prisms are available in^46^. Note, both approximations for self and mutual demagnetization factors may not be adequate for small external fields *B*_0_, as it neglects the potential nonuniform magnetization of GGG.

To ensure that our calculations and numerical micromagnetic simulations would be most precise, the magnetization of GGG *M*_GGG_ was measured for each temperature within the same field range used in the FMR experiments. For calculations at temperatures below 1.8 K, we relied on the VSM data obtained earlier. The data was fitted using Eq. (6) by adjusting the molecular field *λ* = -0.854 and saturation magnetization ${M}_{{{{\rm{GGG}}}}}^{{{{\rm{s}}}}}$ = 831 kA/m as fitting parameters. This fitting method was used to extrapolate the values of *M*_GGG_ with respect to temperature *T* and external magnetic field *B*_0_. It allowed us to compare our theoretical and simulation work with experimental results even at temperatures below 1.8 K.

### Numerical simulations

To gain a better understanding of the magnetic stray field present in the YIG layer on the GGG substrate, we performed numerical simulations that provide a more precise representation of the field inhomogeneity compared to analytical calculations. To simulate the induced stray field in YIG, we solved the magnetostatic Maxwell equations within the GGG layer with a nonlinear material law (e.g. magnetization curves Fig. 2a inset) extracted from the VSM measurements at *T* = 1.8 K. For the simulation a finite element/boundary element method is used with a mesh size of 0.1 mm^47^. The field is evaluated 50 nm above the GGG surface as a projection to the external applied in-plane bias field *B*_0_, as depicted in Fig. 1b.

## Data Availability

The datasets used and/or analyzed during the current study are available from the corresponding author upon reasonable request.
